# Health and Mortality Monitoring in Threatened Mammals: A First Post Mortem Study of Otters (*Lutra lutra* L.) in Italy

**DOI:** 10.3390/ani12050609

**Published:** 2022-02-28

**Authors:** Romina Fusillo, Mariarita Romanucci, Manlio Marcelli, Marcella Massimini, Leonardo Della Salda

**Affiliations:** 1 LUTRIA sas Wildlife Research and Consulting, 00173 Roma, Italy; manlio.marcelli@lutria.eu; 2 Faculty of Veterinary Medicine, University of Teramo, 64100 Teramo, Italy; mromanucci@unite.it (M.R.); mmassimini@unite.it (M.M.)

**Keywords:** Eurasian otter, *Lutra lutra*, post mortem investigations, causes of death, health parameters, road mortality, Italy

## Abstract

**Simple Summary:**

The Eurasian otter (*Lutra lutra*) is a semiaquatic mammal listed as endangered in Italy. We conducted a first post mortem (PM) study of 28 otters, mostly collected between 2009 and 2017, in Italy. Vehicle collision was the most common cause of death and mainly involved young animals. The examined otters appeared to be smaller than individuals from northern Europe, and most of them were in good body condition. However, in some individuals, necropsy exams revealed different signs of disease, such as pleuropneumonia and peritonitis, gastritis, lymphoma, or bacterial infection from conspecific bites. Ticks and endoparasites were rarely detected. Data collected on scene of death helped to identify factors forcing otters to move over the road, suggesting road-kill mitigation measures. Comprehensive, standardized PM investigations through collaborative and coordinated research efforts are essential for otter conservation in Italy.

**Abstract:**

Dead specimens provide valuable data for the conservation of threatened species, allowing investigations of mortality, health conditions, and demographic parameters. The Eurasian otter (*Lutra lutra*) is a semiaquatic carnivore listed as endangered in Italy. In 2009, we started the first post mortem (PM) study of otters in Italy, through collaborative research between mammal ecologists and veterinary pathologists, using standardized protocols. Twenty-eight otters, mostly collected between 2009 and 2017, were examined. Most otters were males (67%), between 1 and 3 years old (64%), and predominantly in good nutritional condition. Adult males were significantly larger than adult females (*p* < 0.02), as expected for the species, although both sexes appeared to be smaller than otters examined in Central–northern Europe. The youngest sexually mature female was 3 years old. Road traffic collisions were the major cause of death, especially in young individuals, and mainly occurred in autumn–winter, particularly for females. Investigations of the scene of death contributed to revealing factors forcing otters to travel out of the water and move over the road, suggesting appropriate measures to reduce vehicle collision risk. Other causes of death included blunt chest trauma of uncertain origin, dog and conspecific attacks, or diseases of infectious or non-infectious origin, such as ulcerative gastritis, pleuropneumonia and peritonitis. Other diagnosed diseases included lymphoma. Ecto- and endoparasites were rarely detected, although we report the first documentation of heartworm and *Ixodes hexagonus* infestation in Italian otters. It is important to continue comprehensive, standardized PM investigations of otters in Italy to define baseline health, biometric and demographic parameters, collect biological samples for comparative analyses, and to reduce road-kill mortality. The present study suggests that the timely collection of carcasses and collaborative and coordinated research efforts are essential for obtaining useful data for the conservation of otters.

## 1. Introduction

The Eurasian otter (*Lutra lutra*) is a semiaquatic carnivore listed as Near Threatened at European and global levels [1] and is considered as endangered in Italy [2]. Once widespread across the country, this mammal is now restricted to the southern regions of the Italian peninsula. The Italian otter population is completely separated from other European populations, despite the fact that a range re-expansion has been reported in recent years and is apparently ongoing [3,4].

In the case of threatened species or populations, dead specimens can provide valuable data to contribute to the evaluation of conservation status, threats, and hypotheses about the causes of declines [5,6]. Gaydos et al. [7] used standardized necropsies of dead killer whales and a disease-testing protocol to determine which infectious agents could have a negative impact on a declining population of this species. In the Eurasian otter, comparisons of tissue levels of contaminants in dead otters collected from healthy and declining populations, or from the same population over time, supported the hypothesis that the bioaccumulation of polychlorinated hydrocarbons, such as organochlorine pesticides (OCs), polychlorinated biphenyls (PCBs), and DDTs, had a relevant role in the decline of European populations during the last century [8,9,10]. 

Post mortem (PM) examination of individuals provides evidence of diseases and causes of death, as well as information on age, sex and reproductive status, which may be used in population modeling and assessment when coupled with the estimation of population sizes (or adequate surrogates) [11].The examination of reproductive systems in female carcasses provides insights about pregnancies, litter size, and the time and seasonality of births, features that are still little-known in most European otter populations [12,13,14].

In general, the examination of dead animals may be the only chance to obtain data on the health status of populations (i.e., the level of contaminants in individuals, evidence of infectious, metabolic, or nutritional diseases, body conditions, and dental health), reproductive success, biometric parameters, risk factors and causes of death, for many wild species, including the Eurasian otter and other threatened taxa. 

Given the relevance of mortality, pathology, anatomical, and ecological data from carcasses for monitoring the health and conservation status of the Eurasian otter, a national scheme for the “reporting-collection-necropsy” of otters found dead has been implemented in the United Kingdom and some other European countries (e.g., Sweden) for decades [15,16,17,18]. During the early 2000s, a coordinated scheme for the collection and PM examination of otter carcasses was still lacking in Italy, although the otter was classified as critically endangered [19]. In an attempt to fill this gap, we started a PM study of otters in southern Italy in 2009. Our aims were: to investigate the health status of the Italian otter population; to describe basic biometric parameters and collect relevant data (i.e., sex, age, and reproductive status) for population modeling and demography; to analyze causes and conditions of death; to start a systematic collection of biological samples for histopathology, chemical, genetic analyses, and future comparisons. The study involved a stable multidisciplinary team including otter ecologists and veterinary pathologists and relied on a standardized, high-quality necropsy procedure and other post mortem protocols designed for the Eurasian otter.

The present paper describes biometric data, sex, age, health, and reproductive status, as well as the causes and conditions of death of 28 Eurasian otters found dead in southern Italy, mainly from 2009 to 2017. 

## 2. Materials and Methods

Otters were submitted for examination and laboratory analyses as part of a collaborative project. Submitters included protected areas staff and law enforcement personnel, wildlife rehabilitators, volunteers of environmental organizations, and members of the general public. When an otter was found dead and reported, a detailed inspection and description of the scene of death was conducted, based on an agreed ad hoc protocol (see Supplementary Material S1). 

Necropsies were performed, and tissue samples, organs, and bones were collected and stored at the Faculty of Veterinary Medicine, University of Teramo, Italy, applying the PM protocol developed by Simpson [20] for the Eurasian otter. Otters were thawed if previously frozen, and sexed, weighed (dry), washed, and measured (nose–anus and anus–tail tip lengths). The external examination of otters included: inspection of the mouth, ears, nose, head and anogenital region, foot and footpads, skin for puncture wounds, other types of lesion and ectoparasites; investigation of dental wear and health, fractures, and visual assessment of nutritional conditions; evaluation of the development and status of mammary glands (e.g., number of protruding suckled teats, presence of milk/colostrum).The internal examination included: dissection and gross pathology of major organs (lungs, heart, kidneys, liver, and spleen), thymus (when present), thyroid and adrenal glands, sub-maxillary salivary glands, and main lymph nodes; analysis of the stomach, gallbladder, and intestine contents; measurement and assessment of reproductive apparatus. The reproductive status of females was assessed based on the size and development of the uterine horns (length > 60 mm in sexually mature females [12]), presence of placental scars or fetuses, nipple size, development of mammary glands, and the presence of milk or colostrum. As far as males were concerned, the length and weight of testes were measured, and undescended (in sexually immature individuals) or descended (in adult males) gonads were recorded.

All organs were weighted to set baseline values for the population. In particular, as far as adrenal glands were concerned, an Adrenal Index (AI) was calculated as the ratio between the cube root of combined glands weight (g) and the body length (m) of the otter [17], in order to evaluate enlargements (for example, due to cortical hyperplasia) as indicators of stress. 

A Condition Index (CI) was calculated for specimens with intact spine by using the following formula: CI = W/*a*L*^n^*, where W = body weight in kilograms and L = nose to tail tip length in meters. The constants *a* and *n* were 5.02 and 2.33, respectively, for females, and 5.87 and 3.39 for males [21]. 

At the necropsy table, otters were aged as cub–juvenile (if one or more deciduous teeth were present), immature (e.g., deciduous teeth absent, body size smaller than adults, and immature gonads), subadult (animals of adult body size with not fully developed gonads and low tooth wear and slender canines) or adult (fully developed gonads and os penis length > 6 cm), using a combination of morphological features. A canine, if available, or a premolar as alternative tooth, was used to determine the exact age in immature to adult otters by counting *cementum annuli* [22]. Each tooth was gently extracted by hand after heating the otter jaw or skull in 70 °C water for 2–3 h. Teeth were cleaned, coded, and stored in individual paper envelopes, and then shipped to the Matson’s Laboratory LLC (135 Wooden Shoe Lane, 59741 Manhattan (Montana), USA) for microscopic analysis of *cementum* rings. Samples of all major organs and tissues were fixed in 10% neutral buffered formalin, processed routinely, embedded in paraffin wax and sections were stained with hematoxylin and eosin (HE). When necessary, additional sections were also subjected to special staining, such as Grocott (for fungal organisms), Alizarin red (for calcium deposits), and Giemsa (for bacteria) stains. Remaining organs and tissue samples were then wrapped in aluminum foils and placed in deep freeze (−80 °C). In particular, samples of liver, skeletal muscle, and fat were frozen for future chemical analyses. Samples of muscle, tongue, or hairs and vibrisse were also placed in 95% ethanol and frozen for future genetic analysis. In addition, all recovered parasites (ecto- and endoparasites) were stored in vials containing 90% ethanol and then identified by parasitologists at the Faculty of Veterinary Medicine, University of Teramo. We used the Mann–Whitney U test for inter-group comparisons and Spearman’s test to evaluate correlations. We applied a chi-square test of goodness of fit to evaluate the hypothesis of equal frequency distribution of road-kills across seasons.

## 3. Results

### 3.1. Numbers, Origin, and Sex of Otters

We examined 26 otters between November 2009 and July 2017. This number represents 46% of dead otters reported at the national level (see data from http://therio.unimol.it:8080/lontra/ (accessed on 28 December 2021)) in the study period. We also considered necropsy findings regarding a male otter that the PM team examined in 2005 and measures and observations from a male found dead in 2019 and examined at the Istituto Zooprofilattico Sperimentale (IZS) of Catanzaro in collaboration with author R.F.

We examined 3.4 otters/year on average. The numbers of otters submitted varied among years (1–6), possibly due to a combination of factors (e.g., the geographic location of deaths within the distribution range of the species and people involved in recovering carcasses).

**Figure 1:**
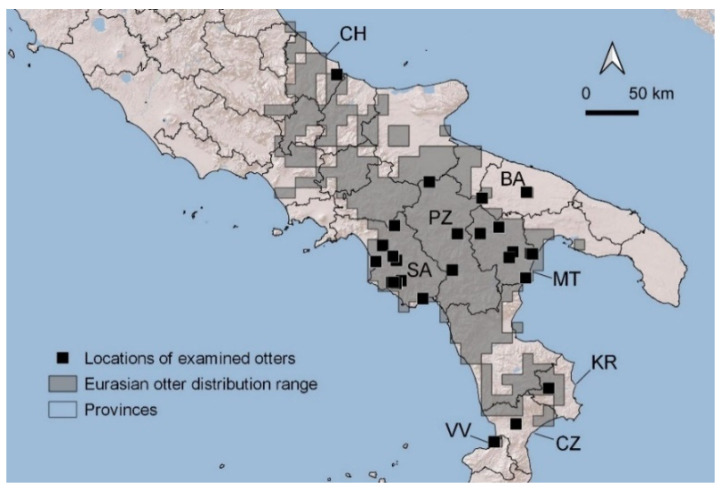
Distribution of collection sites of otters examined during the study. Abbreviations for provinces where otters were found, are showed: CH = Chieti; SA = Salerno; PZ = Potenza; MT = Matera; BA = Bari; KR = Crotone; CZ = Catanzaro; VV = Vibo Valentia.

Overall, the examined otters were from five regions of Italy: Abruzzo, Campania, Basilicata, Puglia, and Calabria. Most of otters came from Matera and Salerno provinces (33 and 37%, respectively). Three specimens came from Potenza province and two from Bari. In the most recent years of the study (2014–2016), single otters were also received from newly recolonized provinces of Chieti, Crotone, and Vibo Valentia (Figure 1). The examined otters were mostly males (67%).

### 3.2. Size, Weight, and Body Conditions

Valid nose-to-anus (NA) and anus-to-tail tip (ATT) measurements were obtained in 15 subadult and adult otters (10 males and 5 females; Table 1), allowing the determination of the total body length (TL). Nose-to-anus length measurement was considered invalid in the remaining 13 otters due to spine fractures. TL and ATT measurements are positively and linearly correlated (V. Simpson unpubl. data). We fitted a linear regression with TL as dependent and ATT independent variables based on valid measurements of males and estimated the total length of specimens with unbroken tails (valid ATT measurement).

Males were larger than females (*p* < 0.02, Mann–Whitney U test) and had average total lengths of 111 cm (range = 103–117, N = 10). The TL of males was still 111 cm on average when estimated TLs were also included (N = 17). The TL was 93–101 cm (mean = 98) in five females with valid NA and ATT measurements. Two juveniles, one female and one male, measured 85 and 88 cm, respectively (Table 1). 

On average, healthy males weighed 6.9 kg (±0.47 standard deviation (SD), N = 12) compared with 4.7 kg (±0.35 SD) for females (N = 5). Males showed CI values ranging from 0.58 to 1.02. The lowest value belonged to a male otter dead for ulcerative gastritis. The mean value of CI was 0.92 in nine healthy males. The range of female CI was between 0.73 and 1.03 and averaged 0.99 (N = 4) when one sick female was excluded (Table 1). 

### 3.3. Age

**Figure 2:**
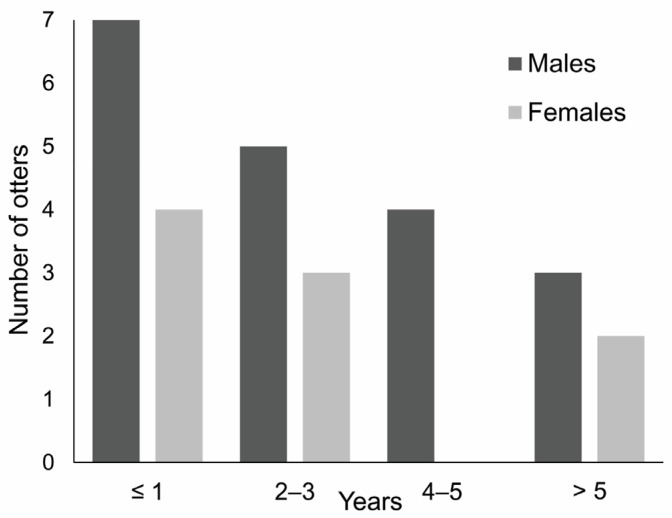
Age distribution of submitted otters.

Otters submitted for necropsy were aged between a few months and 10 years old (Table 1). Thirty-six percent of specimens was ≤1 year (N = 10) and 64% ≤ 3 yrs old. The oldest otter was a male. The age distribution of submitted otters was skewed towards younger individuals in both sexes (Figure 2).

### 3.4. Reproductive Status

**Figure 3:**
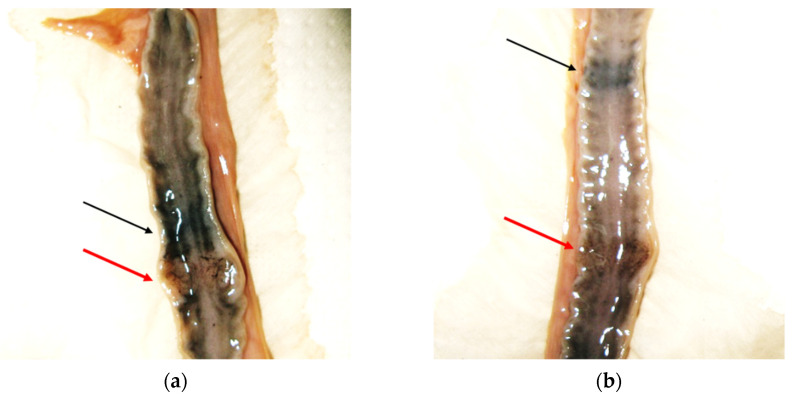
Uterine horns ((**a**) left, and (**b**) right) of a 7-year-old female otter showing placental scars (black arrows) and early placentations (red arrows).

The reproductive status was assessed in six out of nine female carcasses that were in good conditions. Paired uterus length ranged between 37.5 and 62.5 mm, with length ≥ 60 mm in three 3–8 year-old females. Subadult, 1–2 year-old females showed horn lengths between 37.5 and 46.5 mm, which are consistent with those of sexually immature females. In a 7-year-old female, the uterine horns were large (52 and 55 mm), and two placental scars and two early placentations were visible (Figure 3). This female died from natural causes in June and had small nipples, showing no evidence of lactation. 

Nipples were also small in another four females without placental scars or evidence of pregnancy. No sign of reproduction was found in an 8-year-old female. Overall, three females out of six appeared to be sexually mature, although only one showed evidence of pregnancy.

Five males had undescended or partially descended testes. On average, these males were aged 1.02 yrs old. Thirteen males had scrotal testes and were aged 4.31 yrs old on average. Testis length was measured in 10 males with good carcass conditions. Undescended testes measured 14–19 mm (N = 4). Descended testes measured <21 mm in two young, 2-year-old males, and 22–30 mm in four males aged 4.25 yrs old on average (range 1–8 years). Testes weighed 4.60 g on average in seven males with descended testes. A 5-yrs-old male showed testicular asymmetry (left: 3.19 g; right: 2.11 g), with no evidence of pathological conditions.

Fractures or deformity of the os penis were not recorded in the males examined. The length of baculum was significantly correlated with age (Spearman’s rs = 0.7906, *p* < 0.001). Young males, <2 yrs old, had a shorter baculum (mean = 62.36 mm, N = 7) than older males (3–10 yrs old; mean = 69.33 mm, N = 9). 

### 3.5. Causes and Conditions of Death

**Figure 4:**
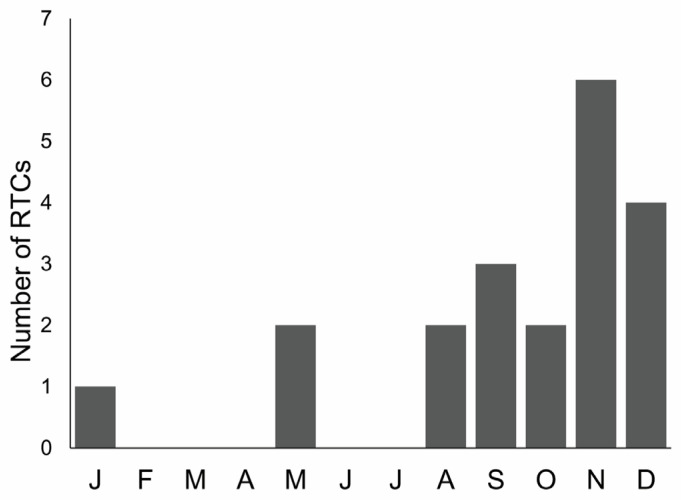
Monthly distribution of otter road-traffic collisions (RTCs) during the study.

The majority (71.43%, N = 20) of submitted otters were killed in road traffic accidents (Table 1), with a higher percentage of females (78%, N = 7) than males (68%, N = 13). In total, 50% of road-killed otters were aged <2 years old, 40% were 2–5 years old, and only two otters were older than 5 years. Road-kills occurred mostly (75%) from September to December (Figure 4), showing a seasonal pattern (Chi-square goodness of fit = 12.3, df = 3, *p* = 0.015; winter = December–February, spring = March–May, summer = June–August, autumn = September–November).

As well, four otters died as a result of cranial (N = 3) or blunt chest (N = 1) trauma of uncertain origin [23]. In addition, one male died from a severe bacterial infection as a consequence of conspecific bite wounding, and another one was killed by dogs. On the other hand, two otters died from pathological conditions, associated with severe emaciation and without evidence of traumatic lesions: one female was affected by fibrino-purulent pleuropneumonia and peritonitis, in association with a reactive, thoracic, and mesenteric lymphadenopathy, whereas one male showed severe ulcerative gastritis (Table 1).

In the case of RTCs, the investigation of the scene of death revealed what may have induced otters to travel out of the water and move over the road. 

Nine out of twenty RTCs occurred between November and January (rainy season) along high-traffic roads, ≤50 m from small culverts carrying ephemeral tributaries or channels under the road, or close to bridges that did not allow the animals to cross via the banks. During rainy seasons, such passages may become temporarily unsuitable for otters because the culvert fills to the top, leaving no air space, or because of high rates of water flow; in such situations, the otters can be forced to leave the watercourse and cross the road. In one case, a weir and embankments blocked the otter passage along the watercourse under the road, thus forcing the animal to cross the road. The scenes examined suggested that the channels or the ephemeral watercourses at RTCs were used by otters to safely move across a reclaimed floodplain or to reach fishponds or watering ponds where anurans congregate. For the above cases, mitigation measures were evaluated and proposed to the relevant authorities (e.g., protected areas). The proposed interventions included an artificial ledge under the bridge with weir and embankments, otter-proof fencing, and dry underpasses in case of small culverts. Such underpasses have to be constructed using 60 to 90 cm cylindrical pipes installed alongside, parallel to the watercourse, and above the maximum water level, with the entrance near the road. This kind of otter passage can be particularly useful where the road passes between different types of habitat resource, such as the main river and fish or watering ponds.

Seven RTCs occurred between May and October in various conditions. Otters were killed along both trunk and secondary roads. The nearest watercourse was in some cases just an impluvium, in other cases a short and small/ephemeral tributary, or the main river or stream. Passages under the roads proved to be suitable for otters, because RTCs did not occur during the raining period. However, all seven animals were aged 1–2 years old and, possibly, they were dispersing individuals that were exploring new territories. 

In the remaining four RTCs, the description of the scene of death did not provide information about the reasons for otters crossing the road.

### 3.6. Necropsy and Histological Findings

Gross examination and histological results were partially influenced by the state of preservation of the carcasses, which varied from moderate to advanced decomposition in some cases, as well as by freezing, which further reduced the diagnostic capacity.

A summary of all gross and histological findings is given in Table 2.

#### 3.6.1. Parasites

*Ticks:* Ticks were found on and around the ears and on the body of three otter carcasses, two males, and one female (all <3 yrs old), submitted in 2010, 2014 and 2016, corresponding to a prevalence of 10.71% among all the examined otters. The infestation intensity appeared to be low, although partial detachment of ectoparasites during transport and storage of the carcasses could not be excluded. Male otters showed only one tick, whereas the female was parasitized by five nymphs and one larva of *Ixodes hexagonus*. 

*Biliary parasites:* Gallbladders in sufficiently good conditions for parasite screening were obtained from 14 otter carcasses. No gallbladder showed thickened walls, and no biliary flukes were recovered.

*Cardio-pulmonary parasites*: An adult heartworm, about 20 cm in length and subsequently identified as *Dirofilaria immitis*, was found in the right atrial chamber of the heart of a 1-year-old, male, road-killed otter, affected by pulmonary adiaspiromycosis [24]. Gross or histological lesions due to dirofilariasis, as well as microfilariae, were not detected. On the other hand, first-stage larvae L1 and eggs of a metastrongyloid were recovered from lung tissue of a 3-yearold male, dead due to severe ulcerative gastritis.

*Intestinal helminthes:* Three adult nematodes of *Contracaecum* spp. were detected in the intestinal tract of a male <2 yrs old found dead along a road, a few kilometers upstream of the mouth of Bradano river (Basilicata region). Eggs of the nematode *Capillaria* spp. were also found in the fecal material obtained from the rectum of the same animal, with a low intensity of infection. Gross examination of the intestine also revealed a moderate and diffuse catarrhal enteritis, possibly as a consequence of nematode parasitism.

#### 3.6.2. External Lesions

*Bite wounds:* Bite wounds consistent with lesions caused by canine teeth of *Lutra lutra* (i.e., 18–22 mm apart) were detected on the head, perineum, and/or feet of four otters (three males and one female). On the other hand, the external examination of the carcass of one male revealed multiple bite wounds on the right side of the body, most likely resulting from a severe dog attack (inter-canine distance: 4.5 cm). In the areas of canine punctures, subcutaneous tissues and adjacent muscles were lacerated, in association with the presence of large ecchymosis. Some penetrating wounds in the thorax with associated rib fractures resulted in severe subcutaneous hemorrhage and hemopneumothorax, likely leading to respiratory failure.

**Figure 5:**
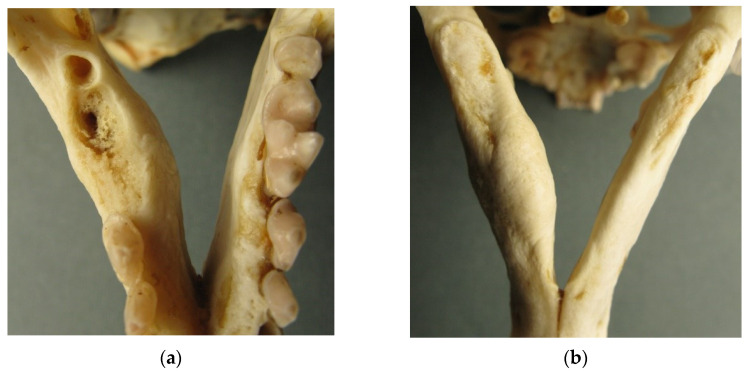
Tooth loss (**a**) and mandibular thickening (**b**) suggesting the presence of osteomyelitis in a 5-year-old male otter.

*Dental lesions:* Forty-six percent of examined animals showed intra vitam dental lesions or injuries. One female and eight males, aged between 1 and 8 years, had fractured canines. Three of these males, respectively 3, 6 and 8 yrs old, also had damaged or missing incisors. Dental malocclusion and caries were also recorded in three and two adult males, respectively. In one of these cases, multiple oral mucosal lesions resulted from irritation caused by dental malocclusion. A male otter <2 yrs old also showed moderate periodontitis around canines and incisors, in association with moderate and diffuse palatal stomatitis. In addition, skull examination of a 5-year-old male revealed maxillary bone resorption exposing upper carnassial (PM4) roots and the almost complete absence of alveolar processes of the lower carnassial, in association with loss of this tooth and mandibular thickening, suggesting the presence of osteomyelitis due to chronic periodontitis (Figure 5).

*Other lesions:* Lead shots were found in leg muscles and perineal tissues of a 1-year-old male. Grossly, shots were not associated with the presence of an inflammatory reaction in the surrounding tissues, and this animal died because of a car accident.

#### 3.6.3. Internal Organs

Complete gross examination of internal organs could not be performed in some cases due to carcass decomposition or severe trauma.

*Cardiovascular system:* Complete detachment and/or traumatic ruptures of the heart and pericardium were seen in several road-killed animals. In addition, left atrial appendage rupture with severe hemopericardium due to blunt chest trauma was observed in one male otter found dead on the roadside [23]. No gross abnormalities other than traumatic lesions were observed in the heart of examined animals. Heart weight was measured in 14 otters (3 females and 11 males) and was related to body weights (Spearman’s rs = 0.8702, *p* < 0.001). Hearts were smaller in females (34–55 g) than males (mean 65 g). The lowest heart weights (34 and 44 g, respectively) were recorded in one female and one male dead in poor body conditions.

**Figure 6:**
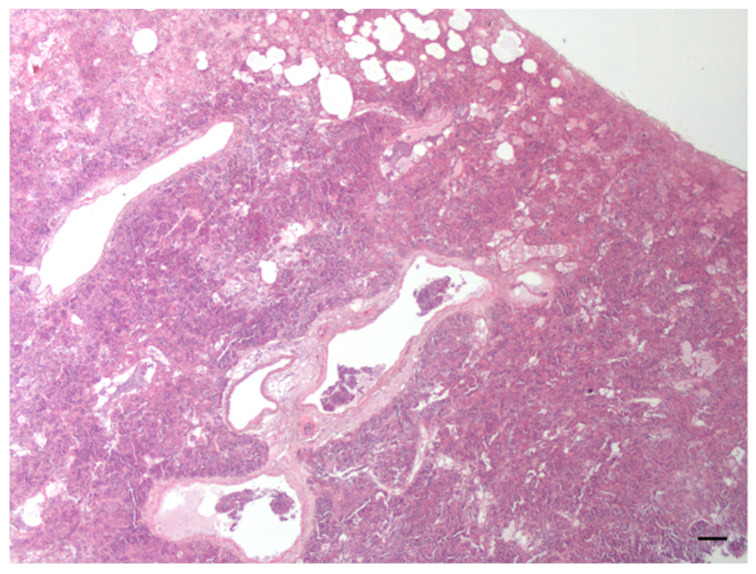
Lung of a female otter affected by severe fibrino-purulent pleuropneumonia: an extensive area of diffuse inflammatory infiltration is visible (HE; bar = 90 μm).

*Respiratory system:* Multiple palpable and/or visible scattered subpleural nodules (≤1 mm) were detected in four male otters. In one case, histological examination allowed a diagnosis of pulmonary adiaspiromycosis to be confirmed [24], whereas in the other three cases, small, frequently mineralized granulomas of unknown etiology were detected. Multifocal areas (≅5 mm) of pulmonary parenchymal consolidation were observed in another male. During necropsy, the dissection of these areas and their observation under a stereomicroscope revealed the presence of nematode eggs and larvae L1 of a metastrongyloid, associated with the presence of multifocal inflammatory infiltrations at histological examination. The female otter affected by pleuritis also exhibited multifocal to diffuse areas of fibrino-purulent bronchopneumonia (Figure 6). In a 6year-old male, a small fragment of *Rubus* spp. sprig as well as pereiopod and uropod of a freshwater shrimp (*Atyaephyra desmaresti* or *Palaemonetes antennarius*) were found within a bronchial lumen, although they were not associated with the presence of macroscopical lesions in the surrounding tissues. 

**Figure 7:**
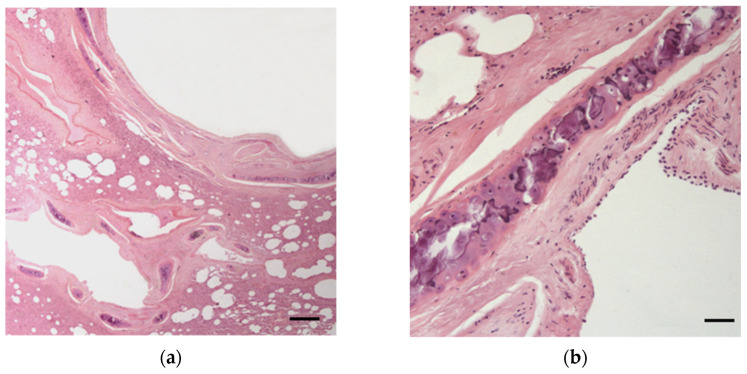
Otter lung showing variably extensive mineralization of bronchial cartilages: the right image shows a higher magnification of the central foci of mineralization (HE; bar = (**a**) 90 μm; (**b**) 25 μm).

Generally, in the histological examination of lungs and respiratory airways, rings of cartilage and a thick muscular wall were observed in the small airways. In addition, tracheal and bronchial cartilages showed the presence of variably extensive central foci of mineralization in all samples examined (Figure 7).

**Figure 8:**
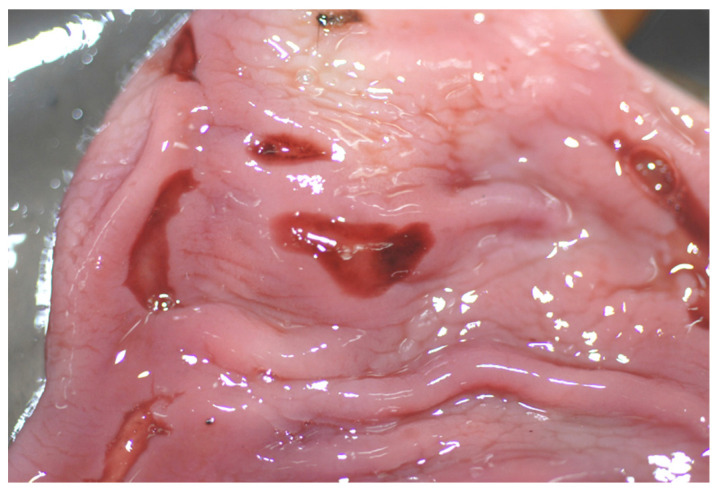
Male otter dead due to severe gastritis: multifocal ulcerated lesions are visible on the gastric mucosa.

*Alimentary system*: Oral or esophageal lesions other than the previously described bite wounds or dental lesions were not detected in the examined otters. In 21 otters, the gastric wall was intact at the time of PM examination. In most of cases (62%), the stomach was empty, sometimes in association with a moderate amount of clear or slightly blood-tinged gastric mucus, or the stomach contained a few prey residuals. Only one male, dead due to severe gastritis, showed multifocal ulcerated lesions on the gastric mucosa (Figure 8), whereas one female, dead due to fibrino-purulent pleuritis and peritonitis, also showed multiple gastric mucosal erosions, in addition to the presence of multifocal areas of adhesion involving the intestinal serosa and parietal peritoneum. In the remaining cases, the most frequently found type of food was constituted by fish (67%, mainly cyprinids, but also eels and brown trout), followed by anurans (33%), invertebrates (25%) and the freshwater crab *Potamon fluviatile* (8%). 

The gut was also examined for the presence of parasites and/or lesions in 21 otters. Intestinal parasites were not detected, with the exception of nematodes of the genus *Contracaecum* observed in one male otter, in association with the presence of moderate and diffuse catarrhal enteritis. 

*Liver and gallbladder:* In 17 of 28 examined otters, the liver was ruptured or pulped. Valid weights were obtained in only two females (147.5 and 215.3 g) and five males (mean = 208 g ± 32.5 SD). The examined livers, including the ruptured organs, did not show abnormalities in gross or histological examinations. The gallbladder was normal in all animals examined, and no parasites were found within the lumen. 

**Figure 9:**
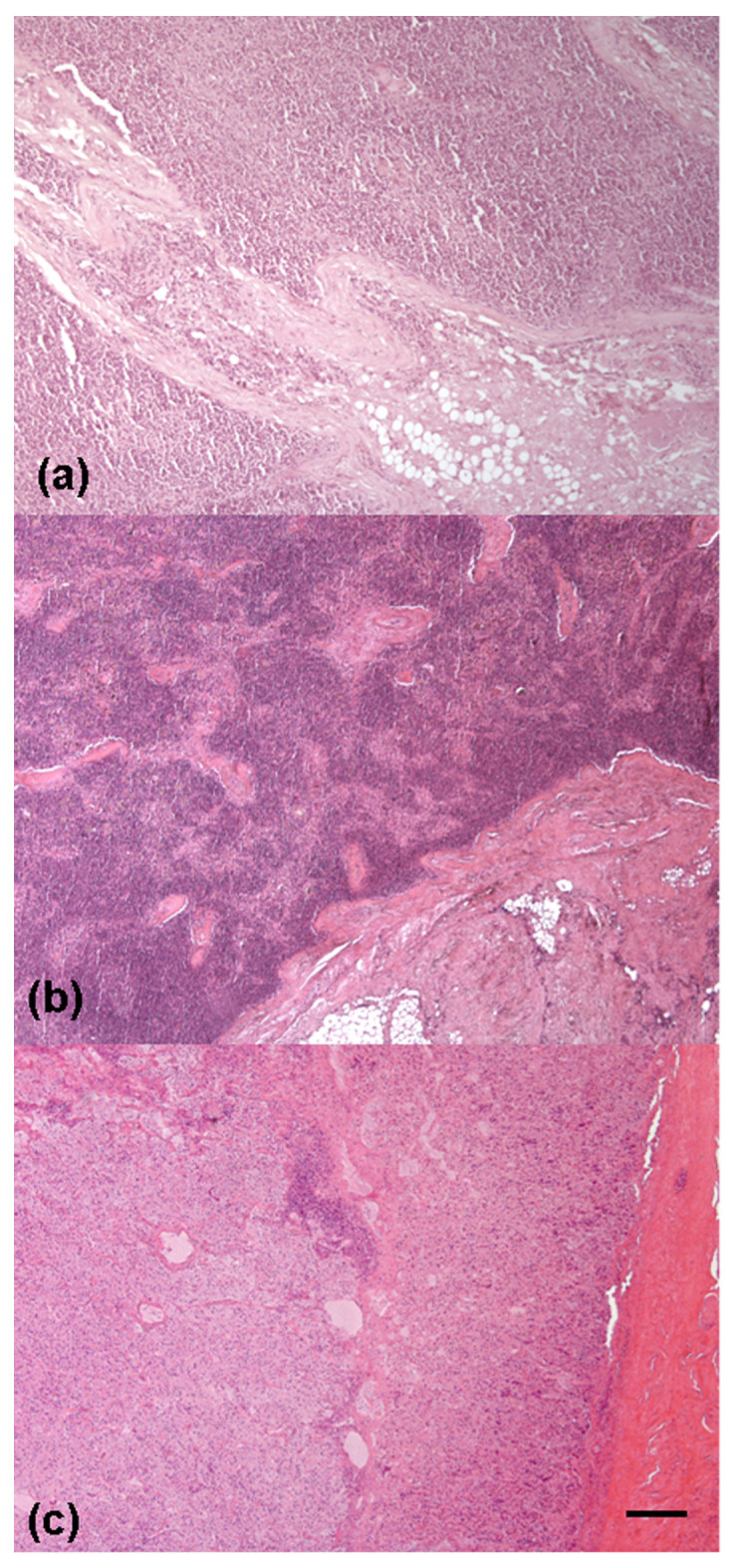
Male otter with multicentric lymphoma: monomorphic population of lymphoid cells invading the lymph node capsule (**a**) and the splenic parenchyma (**b**); (**c**) multifocal, moderate lymphoid infiltrations in the adrenal gland (HE; bar = (**a**) 50 μm; (**b**,**c**) 100 μm).

*Spleen:* The spleen was intact in 2 females and 10 males and weighed 16.3 g and 30.3 g (m ± 7.5 SD) in the two sexes, respectively. No abnormalities were seen, with the exception of a nodular lymphoid proliferation observed in one male, road-killed otter affected by a multicentric lymphoma (Figure 9).

*Urinary system:* Weights of kidneys (valid samples N = 16) ranged from 26.18 to 74 g (total weight of the two kidneys; mean weight ± SD = 39.63 g ± 7.22 in 3 females, and 48.67 ± 13.53 in 13 males) and were significantly correlated with the otter’s age (Spearman’s rs = 0.62, *p* = 0.0105). In some cases in which kidneys were available for histological examination, small multifocal foci of cortical or medullary tubular necrosis associated with dystrophic calcification were detected. 

*Lymph nodes:* As previously described, one female dead due to fibrino-purulent pleuropneumonia and peritonitis showed a reactive lymphadenopathy, characterized by moderate to marked enlargement of thoracic (tracheobronchial and mediastinal) and mesenteric lymph nodes, showing gross and histopathological features of diffuse follicular hyperplasia. In addition, as concerns the male, road-killed otter showing histological features referable to a multicentric lymphoma, the enlarged popliteal and inguinal lymph nodes exhibited loss of cortico-medullary differentiation with the absence of germinal centers. The observed monomorphic population of lymphoid cells also invaded the lymph node capsule and surrounding tissues, a finding that is considered suggestive of malignant lymphoid proliferation (Figure 9).

*Thyroid gland and thymus*: Thyroid size and weight is related to body length in otters. Nevertheless, small thyroids can be found in adult otters found dead in poor health conditions [17]. Due to autolysis or severe trauma, proper thyroid gland examination with valid weighing was obtained for only 10 (2 females and 8 males) otters, without revealing abnormal macroscopical features. Thyroids weighed 0.73 g on average (±0.23 SD). The lowest weights (0.4 g) were recorded in two otters <2 years old with good body conditions. Histological examination of thyroid tissues evidenced a normal, active secretory activity in all cases, in association with the presence of brownish, lipofuscin granules in the cytoplasm of follicular cells in two adult otters. The thymus gland is usually larger in young animals and then regresses in later life. Evaluations for the presence of the thymus gland were only possible for 11 otters. Among these cases, the thymus was present in seven, mostly subadult, otters. As expected, a larger gland was found in young, <2-year-old animals (thymus weight 4.98 g on average, N = 5). Only two adult otters showed the presence of the thymus gland, being 1.47 and 3.96 g in weight. No gross abnormalities were observed in all cases examined. 

*Adrenal glands*: Adrenal glands were found in situ in 15 animals and were weighed (paired weights = 0.6–1.21 g, mean ± SD = 0.47 g ± 0.16) and examined, since enlarged and heavier adrenal glands are expected in stressed and sick otters [17]. Although the AI was slightly >1 in only one healthy, male otter, the adrenal glands of four males and one female grossly showed nodular, usually asymmetrical lesions, histologically characterized by a mild to moderate cortical hyperplasia, frequently associated with the presence of small lymphocyte aggregates at the cortico-medullary junction. In addition, multifocal, moderate to extensive lymphoid infiltrations were observed in the right and left, moderately enlarged adrenal glands of the male otter affected by multicentric lymphoma (Figure 9). 

## 4. Discussion

Our sample of necropsied otters was not large in absolute numbers. The main factor explaining the small number of otter carcasses reported during the study is probably the small size of the Italian otter population, estimated to be <1000 individuals [2]. Indeed, the numbers of otters found dead, particularly road-killed individuals, is positively correlated with the size and trends of the local population, as suggested by some studies [17,25].

The Italian otter population is isolated from other European populations and is considered an evolutionary significant unit (ESU) [26]. Therefore, it is crucial that every otter found dead is promptly submitted for PM analyses. We attempted to achieve this goal through networking with various categories of potential submitters. However, in some cases, the carcasses were not subsequently found after reporting. Moreover, the bodies were often collected several hours or days after death, and/or frozen after collection. These conditions limited the number and quality of PM analyses, in particular histological examinations. Notwithstanding this, our study represented the first comprehensive PM investigation of Italian otters. 

This study provided systematical recording of the body sizes according to a standard measuring protocol [27] and evaluation of the nutritional condition of otters. In general, biometric data of otters are scarce in southern Europe. Based on our data, Italian otters appear to be smaller than otters from Central and northern Europe. Adult otters in Denmark [28,29], the United Kingdom [17,28], Hungary [30], Germany [31], and the Czech Republic [32] were on average 14–33% heavier and between 2–8% longer than Italian otters. Our measures were comparable with those of a study conducted in Spain [33], suggesting a smaller size of otters inhabiting the southern parts of the species range. The geographic variation in otter body size has been explained according to Bergmann’s ecogeographical rule [33,34,35], but it may be also a consequence of the local food availability [34]. We hypothesize that patterns in trophic resources could have favored individuals of small body mass with lower food needs in Italy. Seasonal and inter-annual flow variation of Mediterranean watercourses can determine periods of reduced food (i.e., fish) availability. Accordingly, the diet of otters in southern Europe is less fish-dependent and more based on alternative prey than in temperate areas of Europe [36,37]. Since non-fish prey, such as amphibians and invertebrates, are less nutritive than fish [38], a small body size could be an advantage in exploiting such resources.

The majority of otters submitted for this study were in good nutritional condition. Values of the CI for the healthy individuals examined were just below 1 in both sexes. Simpson [17] considered values ≥1 indicative of a good nutritional status. This slight discrepancy may originate from the larger body size of UK otters, for which the index was developed. 

The age distribution of submitted otters was skewed towards younger individuals in both sexes. A left-skewed age structure was commonly reported in PM studies of otters across Europe [39,40,41,42] (but see [30], where the majority of road-killed otters were aged individuals). Young individuals are less experienced and more prone to die due to traffic collision. The maximum estimated age of 10 years found in this study is comparable to that detected in other studies across Europe [17,43], but see [39].

Based on lengths of uterine horns (>60 mm) and other measurements, Hauer et al. [12] identified sexually mature female otters in Germany. These authors reported 2 years as the age of first reproduction for females, although most of reproductively active females were aged 6–9 years. Our measures of uterine horns >60 mm were observed in female otters 3 years of age and older, suggesting an age of first reproduction for Italian otters similar to that reported in Germany and across Europe. Signs of pregnancy were detected in only one female found dead in June, which showed two placental scars and apparently two recent implantations. Counting of placental scars may be considered indicative of litter size [44]. The most frequent number of detected placental scars was 2 in the UK [14], Hungary [13], and Germany [12]. Our single necropsy finding, as well as occasional observations of otter females with cubs in southern Italy (R. Fusillo pers. obs.), suggest that a litter size of about 2 is also probably common in Italy. The presence of placental scars in uterus can suggest the time of births. In the UK, Simpson [17] and Chadwick and Sherrard-Smith [14] found placental scars during almost every month of the year. Unfortunately, our small sample did not allow us to determine if the same pattern may occur in Italy. However, no placental scars were found in two females found dead in winter. 

Measurements of testes weights obtained in >2-year-old males, found dead during different months, as well as their ratio to body weight (gonado-somatic index of fertility, [13]), suggested a peak of male fertility from late-autumn to spring.

If such variation would be confirmed, a peak of births could occur from spring to summer, when the male fertility reaches its lowest value. Unfortunately, few studies were conducted in this field and mostly concerned temperate Europe [18,45,46,47].

Gross pathology revealed the presence of parasites in few individuals. Four hard tick species of the genus *Ixodes* use *L. lutra* as a host in Europe [48], although both the prevalence and the average infestation intensity are usually low in otters when compared to other carnivores [49]. We found very few *Ixodes* spp. ticks (1–3) per individual, although the detachment of ticks shortly after death cannot be excluded. Prevalence (10%) was similar to that reported in German populations [42,50] but lower than in the UK [48]. The role of otters in the dispersal of tick-borne pathogens and in the maintenance of their sylvatic cycles is still unknown [51], but probably negligible due to habitat specialization and continuous contact with water. In this respect, Santoro et al. [51] provided evidence of infection by rickettsial pathogens in Eurasian otters from southern Italy, although without finding ticks on the otter carcasses. Thus, our report represents the first description of *Ixodes hexagonus* infestation in Italian otters. We detected two nematodes in the cardio-pulmonary system of examined otters, although they were not associated with signs of disease (dirofilariasis or strongyloidiasis): an adult of *Dirofilaria immitis*, as well as eggs and L1 larvae of a metastrongyloid lungworm, possibly *Strongyloides lutrae* (Conboy G. pers. comm.), a fairly common nematode in various species of otters. *D. immitis* has been found in *Lutra lutra* in Southwest (SW) Europe [52] and a recent study in Lithuania proved that otters can act as reservoirs of dirofilariasis in the wild [53]. However, our finding, dated back to 2009, represents the first documentation of *D. immitis* infection of otters in Italy. Parasitic studies in SW Europe suggested that helminth fauna of the Eurasian otter in this area is poorer than in Central and eastern Europe [52], and this could also be the case for otters in Italy. We did not detect gallbladder parasites in our cases, although infections by two trematodes of the family Opisthorchiidae, *Pseudamphistomum truncatum* and *Metorchis bilis*, were recently documented in otter populations across Europe, with variable frequency of occurrence [54]. The diffusion of digenean species in Europe seems to be favored by translocations of fish-stocks or movement of intermediate hosts and parasite eggs with plants, gravel, or water. These activities are probably less frequent in rural and economically marginal areas, such as part of Norway, Scotland, or southern Italy, where gallbladder parasites were not found in otters. We found three adult *Contracaecum* spp. nematodes in a male otter found dead at the mouth of the Bradano river in the Basilicata region. *Contracaecum* spp. (fam. Anisakidae) are parasitic nematodes with a global distribution, using mainly marine fish species as intermediate hosts and aquatic mammals or bird predators as definitive hosts. We speculate that the male hosting *Contracaecum* foraged mainly on marine fish species. This finding represents the first report of *Contracaecum* spp. infection in the Eurasian otter. We found *Capillaria* type eggs, which were also found in feces of otters from Denmark [55], Ireland [56], and Southeast Europe [52] with a variable prevalence.

Adrenal gland hyperplasia found in our examined cases has been previously described by Simpson [15], and it has been associated with stress conditions or PCB intake. In addition, ulcerative gastritis has been described by Simpson [17] in Eurasian otter and has been related to stress factors. Neoplastic lymphoid proliferations, involving mesenteric lymph nodes, thymus, liver, spleen, gastrointestinal, and/or cerebral tissues, have been rarely reported in otters, including sea otter (*Enhydra lutris*) [57,58], Asian small clawed otter (*Aonyx cinerea*) [59], North American river otter (*Lontra canadensis*) [60], and Eurasian otter (*L. lutra*) [61]. In our study, one male, road-killed otter showed histological features of multicentric lymphoma involving multiple lymph nodes, thymus, spleen, and adrenal glands, although freezing of the carcass did not allow for proper immunophenotyping of the lymphoid population. Urolithiasis was not observed in our cases, although nephrolithiasis has been reported in captive and free-ranging Eurasian otters [62]. Notwithstanding this, multifocal, variably intense, renal dystrophic calcifications were histologically detected, possibly resulting from toxic injuries or previous infections, although these lesions were not associated with the presence of inflammatory infiltrations. Different studies analyzed concentrations of mercury and other heavy metals in liver and renal samples from otters [63,64], although information concerning possible, toxic-induced histological lesions were not reported. In this respect, two of the three individuals with renal calcifications showed high concentrations of mercury, copper, and other heavy metal in livers (unpubl. data).

In our study, the respiratory apparatus of all histologically examined otters showed the presence of calcified tracheal and bronchiolar cartilage. Previous studies reported the presence of cartilage in the final airways of sea otters (*Enhydra lutris*) [65], considering these features as an anatomical adaptation of terrestrial animals to water immersion, in order to prevent collapse of the last airways, thus allowing better gas exchanges. However, the consistent presence of calcification areas in the tracheal and bronchial walls described herein, not related to the age of the animals, is an unusual aspect, not reported so far in the literature, that could also represent a peculiar anatomical feature of *Lutra lutra*. In fact, from a functional point of view, it could represent a further reinforcement of the bronchial wall to counteract pressure variations during diving, although other marine species that reach greater depths do not show similar features. In this respect, a circular trachea with partially calcified rings has been described in shallow diving sea (*Enhydras lutris*) and river otters (*Lontra canadensis*), although calcification was not observed in the terminal bronchi [66].

Seventy-one percent of examined otters were victims of road traffic. This percentage is consistent with evidence from larger datasets obtained in Europe and in the Mediterranean basin [41,67,68]. Vehicle collision represents the most recorded cause of otter mortality across Europe [69], but their impact on otter populations is difficult to estimate (but see [70]). Some authors [17,25] reported a positive correlation between the rise of recorded road kills and the increase in population size, or geographic expansion, thus suggesting that traffic could have a minor impact on the survival of healthy populations [71]. In southern Italy, the otter is re-expanding its range [3], and the population is probably increasing in numbers. During the study period, the number of otter road-kills was positively correlated to otter site occupancy (Spearman’s r = 0.8862, *p* = 0.0034) in southern provinces ([3], Marcelli and Fusillo, unpubl. data), supporting the hypothesis that road mortality was density-dependent. In order to prioritize the location of mitigation measures for road mortality, modeling tools have been proposed to identify road sections with high potential for otter fatalities [72,73]. These approaches are useful to suggest where implementation is necessary, but not which mitigation measures can be implemented. Our study showed that in several cases, a detailed description of the scene of death may help us understand why otters leave the watercourse and move on the road, enabling a focused design of mitigation measures. For this reason, we recommend including the description of the scene of death as a standard in otter PM protocols. Road mortality showed a seasonal pattern with most otter road-kills occurring during autumn and winter. A similar pattern has been recorded in other otter PM studies [15,17,42,70,72] and has been related to heavy rains and floods occurring in autumn–winter, making swimming through culverts difficult for otters [15,72]. It has been also hypothesized that otters are more vulnerable to road casualties during autumn–winter, because the darkness is prolonged and a large overlap between otter and human activities, including driving, occurs [17,72]. In our study, all road-killed females were collected during rainy seasons. Females were significantly smaller than males and probably less capable of swimming in floods and fast-flowing waters. Thus, they could leave watercourses more often than males in fall–winter seasons, becoming more prone to fatalities. Since focusing on female mortality is vital for threatened otter populations, PM research should specifically investigate road mortality in females and identify actions to reduce its impact. However, road-killed otters were mostly 1–2-year-old males in our cases. A male bias in road fatalities has been extensively reported in otter and mustelids studies [41,42,72,74], and it could be a consequence of larger home ranges, higher energetic requirements, or long distance traveled [37,41,75]. Moreover, otters less than 2 years old are often dispersing individuals that move extensively and may result in being more prone to fatalities.

Three otters died from natural causes (11%). Other studies in Central and northern Europe reported similar or lower percentages of recorded natural deaths [29,41,42]. Obviously, causes of natural mortality detected in submitted death otters do not reflect the rate of natural mortality in Eurasian otter populations (an otter dying from natural causes far away from roads is rarely detected). The probability of finding dead specimens may vary up to 40 times among different causes of death [76]. Thus, different probabilities of detecting a certain cause of mortality introduce relevant bias. Nonetheless, non-violent deaths are essential for improving the knowledge on otter diseases. Efforts should be made to increase the sample of natural deaths, for example, through an active, systematic search along selected watercourses of a study area.

No evidence of deliberately killed otters was recorded in our study. Contrary to countries in Central or SW Europe, where the otter is considered a pest or a competitor by fish-farmers and anglers [77,78,79], illegal killing of otters is probably rare in southern Italy. Aquaculture production and sport fishing are not economically important in southern Italy; thus, reasons for human–otter conflict do not exist. The low potential for human–otter conflict represents a favorable premise for the conservation of *L. lutra* in our study area, and for engaging the general public in carcass collection and PM studies (Supplementary Material S2). However, human-related mortality may occur unintentionally through events such as hunting, net-fishing, or dog attacks. Otters can be accidentally shot, as suggested by the finding of lead shots in a male otter, especially when hunting is practiced along rivers, as in our study area. Dog wounds were immediately fatal for one of the examined otters, a male with good body conditions. Dog attacks have been reported as cause of death in *L. lutra* worldwide [17,41], although at a very low rate and mostly involving cubs and emaciated individuals. Since the male was found dead in a cultivated area with scattered point aquatic habitats, it may be hypothesized that long distance traveling in an open area favored vulnerability to dog attacks. Conspecific bite wounding could be a non-negligible cause of death in Eurasian otter. We recorded otter bites in 14% of cases, although bites represented an indirect cause of death in only one case. Simpson [80] found increasing numbers of otters with conspecific bites in increasing otter populations in Wales.

## 5. Conclusions

Post mortem analyses of dead specimens provide useful data to assess the conservation status of threatened mammals and the efficacy of conservation efforts. We implemented and attempted to make efficient a scheme for the collection and examination of otter *L. lutra* carcasses in Italy, through collaborative research between otter ecologists and veterinary pathologists and the use of standard PM protocols. Our experience showed the importance of the proven and timely collection of bodies in order to avoid data loss and increasing the availability of fresh carcasses, needed for the meaningful interpretation of pathological lesions. This goal can be achieved by creating a stable network of volunteer-based organizations and trained people (training should include health and safety procedures for handling and removal of carcasses), authorized to the collection and transportation of otters found dead. It is vitally important to continue PM monitoring of otters in Italy. In particular, we urge a systematic assessment of legacy and emergent contaminants body burdens in otters found dead and the establishment of a national tissue bank. The availability of banked biological samples will allow proper monitoring of otters’ exposure to environmental pollutants and biomonitoring of contaminants in freshwaters, morphology studies, histology and histopathology investigations, immunohistochemical, as well as molecular analyses, and emerging research applications such as microbiome studies. In recent years, a number of scientific papers focusing on single necropsy findings of otters have been published in Italy. However, we stress the need of comprehensive, standardized PM investigations, including measurements and sample collections, and of collaborative and coordinated research for the conservation of *L. lutra* in Italy.

## Figures and Tables

**Table 1:** Locality and date of collection, sex, age, body size, condition index (CI), and cause of death of examined otters. Only valid weights and total lengths (TLs) are shown. Ages in years are based on count of cementum rings. Causes of death: RTC = road traffic collision. Province: CH = Chieti (Abruzzo region); BA = Bari (Puglia region); SA = Salerno (Campania region); PZ = Potenza, MT = Matera (Basilicata region); CZ = Catanzaro, KR = Crotone, VV = Vibo Valentia (Calabria region).

Animal Code	Collection Date	Locality	Province	Sex	Weight (kg)	TL (cm)	CI	Age	Cause of Death
M1	08/01/2005	Serre	SA	Male	7.20	-	-	5	Consequence of otter bites
M2	18/10/2009	Salento	SA	Male	6.80	117	0.7973	1	RTC
M3	10/04/2010	Aquara	SA	Male	4.59	114	0.5781	3	Ulcerative gastritis
M4	05/09/2011	Castelnuovo C.	SA	Male	-	-	-	1	RTC
M5	28/09/2012	Valva	SA	Male	-	-	-	10	RTC
M6	25/09/2012	Grumento Nova	PZ	Male	5.88	103	0.9350	1	RTC
M7	09/12/2012	Melfi	PZ	Male	7.20	112	0.9412	2	RTC
M8	21/01/2013	Vallo di L.	SA	Male	7.20	113	0.9174	5	RTC
M9	12/01/2010	Policoro	MT	Male	3.11	89	-	Juvenile	Other trauma
M10	05/11/2012	Pisticci	MT	Male	-	-	-	Adult	RTC
M11	08/05/2013	Pisticci	MT	Male	7.35	-	-	3	RTC
M12	25/11/2013	Bernalda	MT	Male	4.8	107	0.6906	Immature	RTC
M13	06/04/2014	Policastro B.	SA	Male	7.10	113	0.9047	6	Other trauma
M14	28/05/2014	Matera	MT	Male	-	-	-	1	RTC
M15	30/08/2014	Cupello	CH	Male	6.70	105	1.0175	1	RTC
M16	22/09/2014	Pizzo Calabro	VV	Male	7.40	115	0.9042	8–9	Other trauma
M17	28/03/2016	Gravina di P.	BA	Male	6.40	105	0.9719	5	Dog attack
M18	27/08/2017	Belvedere di S.	KR	Male	6.40	-	-	Subadult	RTC
M19	11/11/2019	Marcellinara	CZ	Male	7.12	114	0.8940	Adult	RTC
F1	08/11/2009	Vallo di L.	SA	Female	4.5	-	-	8	RTC
F2	15/12/2009	Capaccio	SA	Female	5	99	1.0196	Subadult	RTC
F3	28/06/2010	Felitto	SA	Female	3.8	101	0.7348	7	Fibrino-purulent pleuropneumonia and peritonitis
F4	14/11/2011	Calciano	MT	Female	4.47	100	0.8904	1	RTC
F5	12/01/2010	Policoro	MT	Female	2.78	88	-	Juvenile	Other trauma
F6	20/11/2013	Bernalda	MT	Female	-	-	-	1	RTC
F7	14/12/2014	Brindisi di M.	PZ	Female	5.1	99	1.0327	1	RTC
F8	19/12/2014	Metaponto	MT	Female	-	-	-	3	RTC
F9	14/10/2015	Cassano delle M.	BA	Female	4.3	93	1.0272	2	RTC

**Table 2:** Summary of gross and histological findings recorded in 28 otters found dead in southern Italy.

PM Examination	Findings	Number of Cases
Ectoparasites	*Ixodes* spp.	3
Cardio-pulmonary parasites	*Dirofilaria immitis* adult	1
	*Metastrongyloid* larvae L1	1
Intestinal helminthes	*Capillaria* spp. eggs	1
	*Contracaecum* spp. adults	1
External lesions	Otter bite wounds	4
	Dog bite wounds	1
	Lead shots	1
Dental and oral lesions	Fractured canines	8
	Damaged/missing incisors	3
	Dental malocclusion	3
	Mucosal lesions associated with malocclusion	1
	Dental caries	2
	Periodontitis and palatal stomatitis	1
	Suspected osteomyelitis with bone loss due to chronic periodontitis	1
Cardiovascular system	Left atrial appendage rupture	1
Respiratory system	Adiaspiromycosis	1
	Mineralized granulomas (unknown etiology)	3
	Fibrino-purulent pleuropneumonia	1
	Foreign material without lesions	1
	Mineralization foci in tracheal and bronchial cartilages	13
Alimentary system	Ulcerative gastritis	1
	Erosions of gastric mucosa	1
	Chronic peritonitis with multifocal adhesions	1
Urinary system	Multifocal tubular necrosis with dystrophic calcification	3
Spleen	Nodular lymphoid proliferation	1
Lymph nodes	Multicentric lymphoma	1
	Reactive lymphadenopathy	1
Endocrine system	Nodular adrenal hyperplasia	5
	Lymphoid infiltrations in enlarged adrenal glands	1

## Data Availability

The data presented in this study are available on request from the corresponding authors.

## Supplementary Materials

The following supporting information can be downloaded at: https://www.mdpi.com/article/10.3390/ani12050609/s1, S1: Protocol for the description of the scene of death; S2: Vademecum for reporting mortality events of otters.
