# Supplementary material for: Health and Mortality Monitoring in Threatened Mammals: A First Post Mortem Study of Otters (Lutra lutra L.) in Italy
Source: Animals (Basel). 2022 Feb 28;12(5):609. doi: 10.3390/ani12050609 (PMC8909196; doi:10.3390/ani12050609)
Supplement: Supplementary file 1 [file animals-12-00609-s001.zip › Supplementary material/S1_Protocol_for_reporting_scene_of_death__.pdf]

Protocol for the description of the scene of death

ID (a progressive number identifying the otter carcass)...................

Locality .... Municipality ....

UTM Coordinates...

Date and time ....

Weather conditions ....

Weather conditions (recent days)..

Environment temperature (° C)...

A. Carcass conditions

State of decay

Fresh carcass (no visible swelling; no odours; eyes are not yet dehydrated/opacified; null or few fly eggs around nose, ears, anus, etc) ☐

Early decomposition/bloating stage ☐

Advanced decomposition (*colliquative stage/black putrefaction to butyric fermentation)* ☐

Dry decay/skeletonization ☐

Rigor mortis (full 24-30 h; resolving > 60 h during winter-spring in Southern Italy) Yes/No

Carrion insects

**Blowfly eggs**	Yes/No		
How many?	Where on the body?		
**Blowfly instar**	Yes/No		
**Blowfly pupae**	Yes/No	**Are larvae/pupae collected?**	Yes/No
**Necrophagous coleopteran**	Yes/No		
**Coleoptera larvae**	Yes/No	**Are insects/larvae collected?**	Yes/No

Other

**Presence of other otter/wildlife carcasses around**	Yes/No
**Evidence of animal feeding upon the carcass**	Yes/No
**Presence of bait or partially eaten prey**	Yes/No

Bait/partially eaten prey description ....

Evidence of a traumatic event (other than vehicle collision)...

Position of the carcass ....

B. Finding location

Nearest water habitat:

stream/river	channel	Impluvium/ephemeral watercourse	pond	lake/reservoir

Are there **fishfarms or ponds** that could be attractive to otters, in the surroundings? Yes/No

If so, provide a brief description ....

Name of the nearest watercourse: ....

Watercourse riverbed (m): .... **Flow conditions:** ....

Is the river overflowing? Yes/no

Bank height (m):

≤0.5	>0.5-2	>2-5	>5-10	>10-20

Bank profile:

gentle or composite	steep (>45°)	vertical

Bank modification:

reprofiled	reinforced (top/total/base)	artificial	embanked

Bank vegetation:

bare bank	short herbs or grasses	scrub or shrub	saplings and trees

Presence of artificial features:

weir (m).............	sluice	bridge	culvert	outfall/intake

Other (please specify)..

Pictures of artificial features ☐

Bridge description: height (m)............................... with ☐ or without ☐ central piers

With ☐ or without ☐ bank abutment

Is there bank space that will be not submerged in the event of flooding, under the bridge? Yes/No

Culvert description: shape..............................width (m)..............multiple culverts? Yes/No

Pictures of bridge/culvert ☐

Evidence of recent management or works:

dredging	bank mowing	riparian vegetation cutting	enhancement
sand-gravel extraction		river rehabilitation	other

Other (please specify)...

Presence of fishing tools (e.g. illegal fishnet, fishtraps) Yes/No

Distance (m) of the carcass to the watercourse.................**and to the bridge/culvert**..................

Distance (m) of the carcass to attractive habitats/fishfarms in the surroundings.........................

Name/Code of the nearest road or of the road of the otter RTC…………………………………………………

Type of roads:

Motorway (A or E)	high- speed highway (SS)	trunk road (SS)
secondary road (SP)	paved secondary/rural road	other

Other (please specify)………

Number of lanes: …………… **Speed limits (km/h):** ……………….**Total width:** ……………………………….

Traffic (high/medium/low): ……………………………………………………………………………………………

Presence of fencing Yes/No **Road lighting** Yes/No

Surroundings:

urban area	rural area	natural area

Pictures of the road ☐

Distance (m) of the carcass to the road………………**and to the bridge/culvert**……………….

Position of the carcass on the road……………………………………………………………………………

Identification of possible pathway (otter trail) from the watercourse to the road Yes/No

Pictures of the trail ☐

Please, map the relative position and distances of the watercourse, road, attractive habitats (if any)

(example)

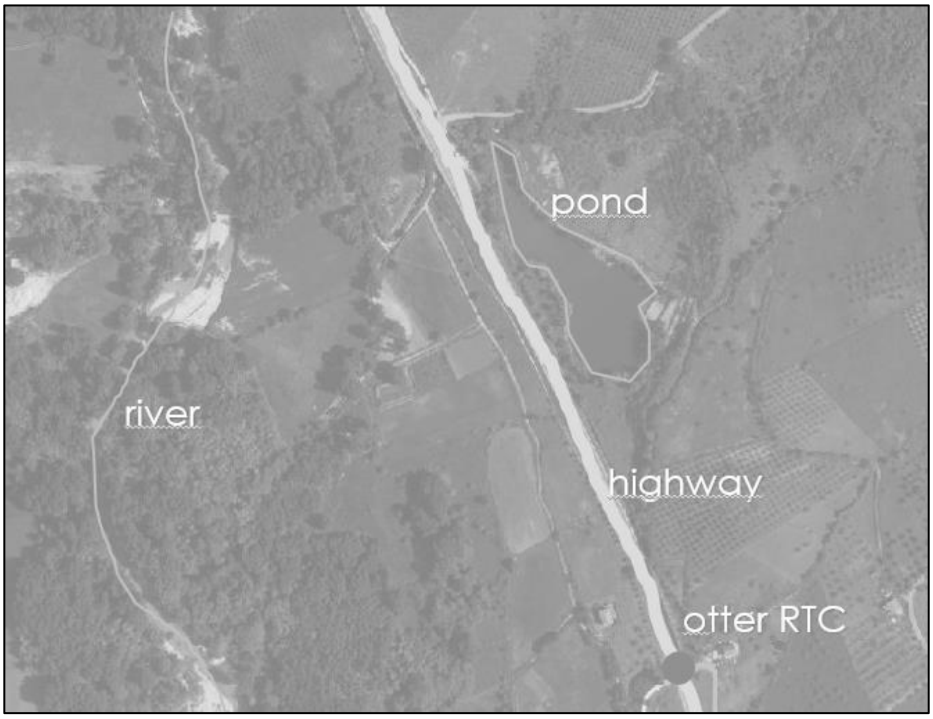

General notes……

……

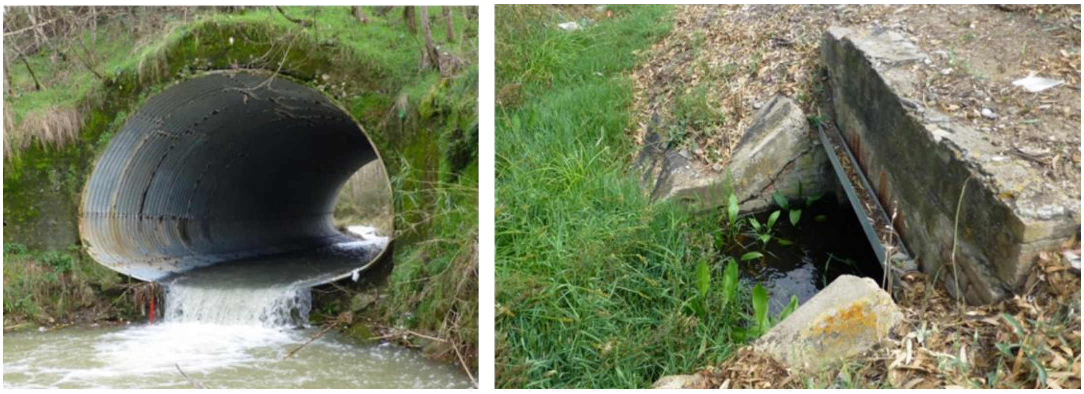

Examples of unsuitable culverts at otter RTCs in Southern Italy

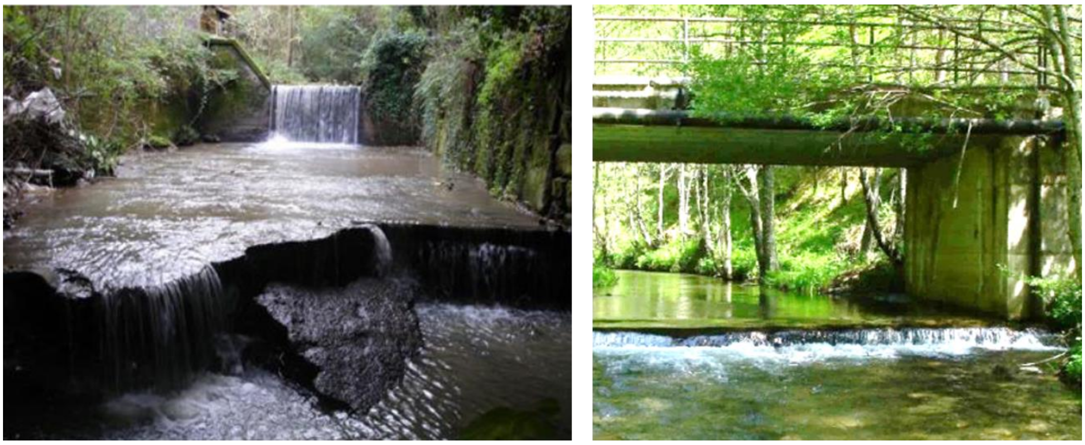

Example of a weir impossible to overcome for an otter at an otter RTC in Southern Italy (left); example of bridge without bank abutment (low suitability during flooding) (right).
