# Supplementary material for: Health and Mortality Monitoring in Threatened Mammals: A First Post Mortem Study of Otters (Lutra lutra L.) in Italy
Source: Animals (Basel). 2022 Feb 28;12(5):609. doi: 10.3390/ani12050609 (PMC8909196; doi:10.3390/ani12050609)
Supplement: Supplementary file 1 [file animals-12-00609-s001.zip › Supplementary material/S2_Vademecum_reporting_otter_mortality_events_IT.pdf]

What to do in the event of finding a dead otter
(for general public)

Premise

The Eurasian otter (*Lutra lutra*) is a semi-aquatic carnivore of conservation concern. This species is strictly protected under the Italian law n. 157/1992. The Eurasian otter is a species of EU interest and is listed in annexes II and IV of the Habitats Directive 92/43/CEE. It is also listed in Annex II of the Berne Convention on the Protection of European Wildlife and Natural Habitats, and in the appendix I of the Washington Convention (CITES).

Given its status of protected species, it is prohibited to disturb, capture, kill, take from the wild, keep or transport live otters (article 12, Habitats Directive; specific derogations for research purposes can be obtained by the Ministry of Ecological Transition). These provisions do not apply to dead animals (see Mò *et al.* 2013). Wildlife carcasses can be used for research purposes under no special condition or derogations, when "*entire bodies or parts of wild animals, other than wild game, are not suspected of being infected or affected with a disease communicable to humans or animals*" (Regulation (EC) No 1069/2009 of the European Parliament and of the Council of 21 October 2009). The Health authority is responsible for assessing the condition, for a dead wild animal, of being or not, infected or affected with a zoonotic disease. Therefore, the collection of an otter carcass requires the intervention of a veterinary health officer from the Local Veterinary Service (LVS), which will evaluate the above mentioned condition. LVSs belong to the Italian Public Health System for Animal Health and Food Safety. Upon request, the LVS may authorize the carcass submission for post-mortem analyses to Universities or research groups/institutes involved in otter research projects, under delivery or transportation conditions which ensure proper safety and healthy protocols. In absence of a specific request for research purposes, the LVS will routinely send the otter carcass to the territorially competent Istituto Zooprofilattico.

Who to call

- General emergency number 112 or 1515 (environmental emergencies)
- Local Veterinary Service (ASL Servizio Veterinario)
- Provincial Police
- Nearest wildlife rescue center (CRAS) or protected area
- Otter specialists and researchers, environmental organisations

When calling the emergency number 112 you can ask to speak to the nearest station of the Comando Carabinieri per la Tutela della Biodiversità e dei Parchi belonging to CUTFAA - Comando Unità per la Tutela Forestale, Ambiente e Agroalimentare. This special Carabinieri corps is devoted to the protection of biodiversity.

Carabinieri, CRAS, protected areas, otter specialists or volunteers of main environmental organizations, are informed about the procedure for the collection of the carcass and will immediately contact the Local Veterinary Service (LVS).

If the phone number is available to you, you can directly contact the Local Veterinary Service (LVS).

What to do or not to do, what to observe and record

A. Be sure that the animal you are observing is an otter

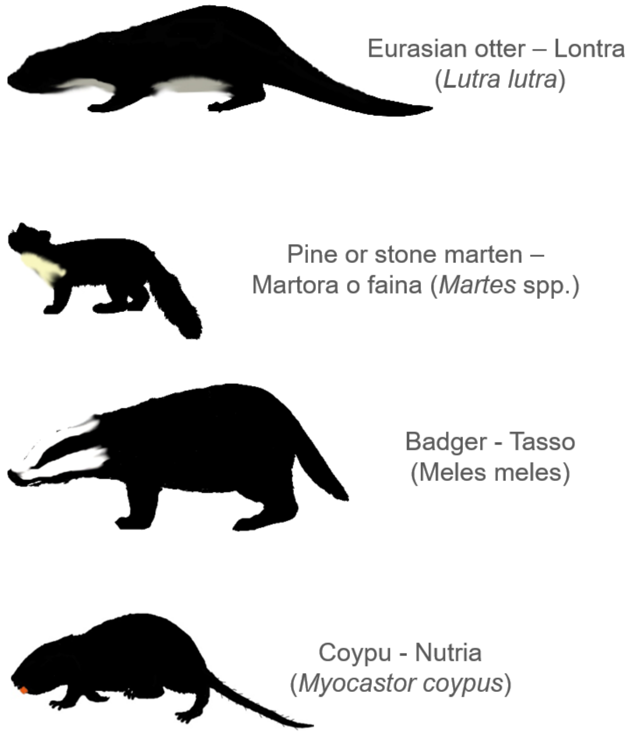

Observing the silhouette and the size of the carcass can help identifying an Eurasian otter. The total body and tail length of an adult otter is about 1-1.2 m. The tail is about 40 cm long. However, cubs or juveniles are usually <70 and <90 cm long, respectively. The fur, dense and short, is brownish-gray, paler on ventral part of the body. The tail is haired. The otter is a carnivore. If you see large orange-yellowish incisors, the dead animal you are observing is probably a coypu. Look at the tail. The tail is almost bare in the coypu , with only sparse hairs. The coypu is smaller than an otter.
Martens are smaller (total body and tail length about 60-70 cm) than an otter and show yellowish-orange (pine marten) or white (stone marten) throat. The badger is the largest mustelid living in Italy. However its tail is short, the body is not elongated as in the otter, and has a distinctive white and black facial mask.

B. Do not touch or handling the carcass

Avoid touching or handling the body. However, if the otter is on a road, and only if you believe it is safe, you could move the carcass toward the margin of the road by a stake or other tool, in order to avoid further damages to the body (this is important for recording valid measurements and weights and for organs examination and collection).

C. Record date and time and coordinates

You can use your mobile device to record the exact coordinates of the finding location. Switch on the location service on your mobile, then open the Google Maps app, find your place on the map, then touch and hold to drop a pin. Google Maps will then display the latitude and longitude of your location.

D. Call the numbers listed above and, if it is possible, wait for arrival of the LVS veterinary officer

During the call, describe the finding location and communicate the coordinates.

E. Take pictures and describe the location and the body

Us your mobile device to take one or more "landscape" pictures of the location and one or more pictures of the body and its relevant details. If possible, provide a brief description of the finding location and conditions.

- Record the name/type of the road (in the event of a road-kill)
- Record the name/kind of the nearest watercourse and other water habitats in the vicinity of the carcass (for example small channels, ponds, impluvia)
- Record the presence of a bridge or a culvert in the vicinity of the carcass
- Record if it is raining and/or it was raining during recent days
- Record if river overflowing is occurring
- Detail where the carcass has been found (position along the road or elsewhere)
- Observe and record if the otter carcass is fresh in your opinion (e.g. eyes are not yet dehydrated/opacified; no visible swelling; no odours; null or few insects and fly eggs around nose, ears, anus etc)
- Record the approximate length of the body
- If you can see the genital area, try to determine and record the sex following the scheme below

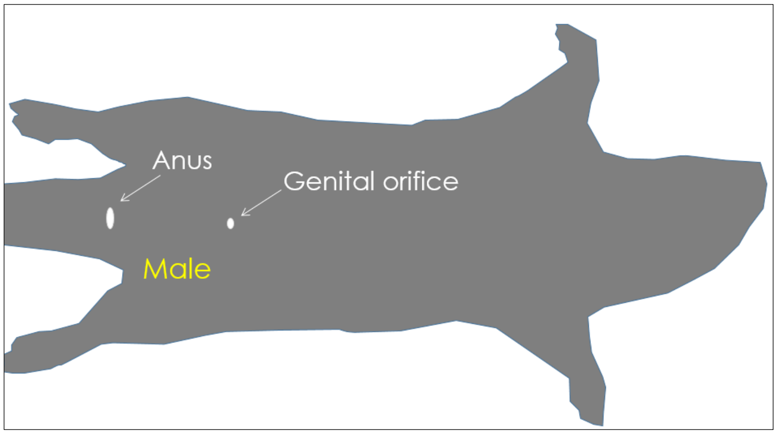

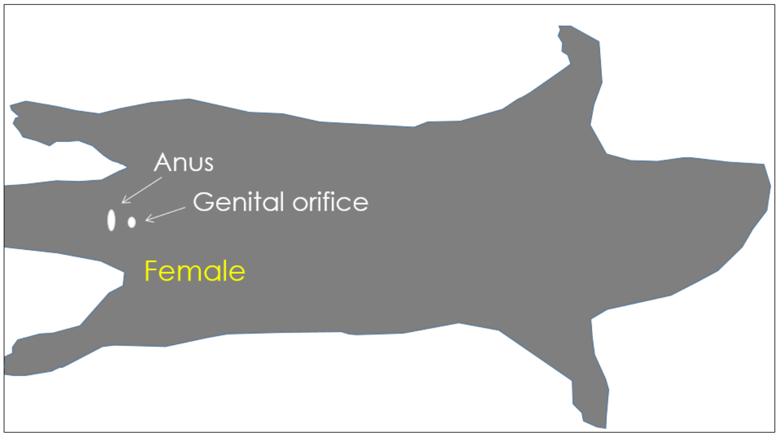

F. Share your information with otter conservationists and the otter community

You can report your finding here:

- lontra-list@googlegroups.com
- https://www.facebook.com/lontraitalia/
- grimec@mammiferi.org (GRiMeC is part of the Italian Mammal Society and is an expert group for the study, conservation and management of Italian mesocarnivores, including the Eurasian otter).

Your information will be included in the map of otter findings at http://therio.unimol.it:8080/lontra/

For authorized personnel responsible for the collection of the otter carcass:

Avoid to pick the carcass up by the tail. Try to gentle roll up the body before putting it into a bag or a sack.

If the carcass is fresh, it should be not frozen and the necropsy should be done asap. Avoid using plastic bags. For transportation put the carcass into a jute sack and then in a cooler box. For temporary storage in the refrigerator, put the carcass within the jute sack, in a rectangular basin.
